# Chelation-induced diradical formation as an approach to modulation of the amyloid-β aggregation pathway†
†Electronic supplementary information (ESI) available: Details of experimental procedures and supplementary figures and tables. See DOI: 10.1039/c4sc01979b



**DOI:** 10.1039/c4sc01979b

**Published:** 2014-10-30

**Authors:** Meghan R. Porter, Akiko Kochi, Jonathan A. Karty, Mi Hee Lim, Jeffrey M. Zaleski

**Affiliations:** a Department of Chemistry , Indiana University , Bloomington , Indiana 47405 , USA . Email: zaleski@indiana.edu; b Department of Chemistry , University of Michigan , Ann Arbor , Michigan 48109 , USA; c Department of Chemistry , Ulsan National Institute of Science and Technology (UNIST) , Ulsan 689-798 , Korea . Email: mhlim@unist.ac.kr; d Life Sciences Institute , University of Michigan , Ann Arbor , Michigan 48109 , USA

## Abstract

Current approaches toward modulation of metal-induced Aβ aggregation pathways involve the development of small molecules that bind metal ions, such as Cu(ii) and Zn(ii), and interact with Aβ. For this effort, we present the enediyne-containing ligand (*Z*)-*N*,*N*′-bis[1-pyridin-2-yl-meth(*E*)-ylidene]oct-4-ene-2,6-diyne-1,8-diamine (**PyED**), which upon chelation of Cu(ii) and Zn(ii) undergoes Bergman-cyclization to yield diradical formation. The ability of this chelation-triggered diradical to modulate Aβ aggregation is evaluated relative to the non-radical generating control pyridine-2-ylmethyl-(2-{[(pyridine-2-ylmethylene)-amino]-methyl}-benzyl)-amine (**PyBD**). Variable-pH, ligand UV-vis titrations reveal p*K*_a_ = 3.81(2) for **PyBD**, indicating it exists mainly in the neutral form at experimental pH. Lipinski's rule parameters and evaluation of blood–brain barrier (BBB) penetration potential by the PAMPA–BBB assay suggest that **PyED** may be CNS+ and penetrate the BBB. Both **PyED** and **PyBD** bind Zn(ii) and Cu(ii) as illustrated by bathochromic shifts of their UV-vis features. Speciation diagrams indicate that Cu(ii)–**PyBD** is the major species at pH 6.6 with a nanomolar *K*_d_, suggesting the ligand may be capable of interacting with Cu(ii)–Aβ species. In the presence of Aβ_40/42_ under hyperthermic conditions (43 °C), the radical-generating **PyED** demonstrates markedly enhanced activity (2–24 h) toward the modulation of Aβ species as determined by gel electrophoresis. Correspondingly, transmission electron microscopy images of these samples show distinct morphological changes to the fibril structure that are most prominent for Cu(ii)–Aβ cases. The loss of CO_2_ from the metal binding region of Aβ in MALDI-TOF mass spectra further suggests that metal–ligand–Aβ interaction with subsequent radical formation may play a role in the aggregation pathway modulation.

## Abstract 2

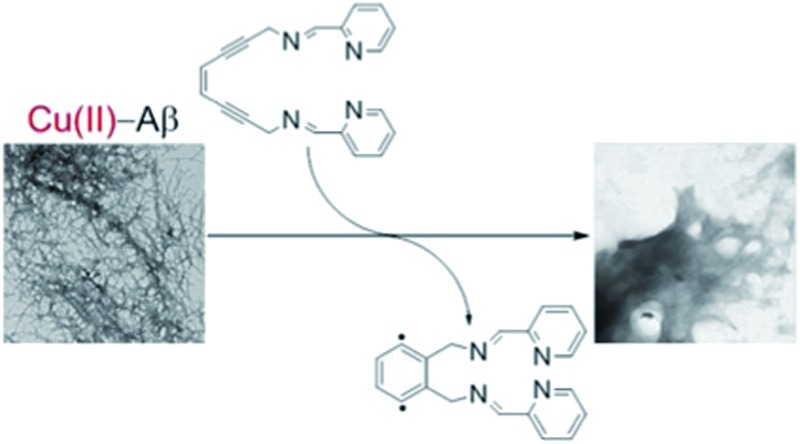
We demonstrate that ligand–metal–Aβ interaction with subsequent radical generation is a relatively rapid mechanism for influencing Aβ structural integrity and thus, the aggregation pathway.

## Introduction

Alzheimer's disease (AD) is the most common form of dementia, affecting over 24 million people worldwide.^1^ It is estimated that this number will nearly double by 2030, partly due to demographic aging resulting from improved healthcare.^1^ The disease presence and progression are pathologically characterized by accumulation of misfolded amyloid-β (Aβ) peptides deriving from β- and γ-secretase cleavage of the amyloid precursor protein (APP)^2–4^ to produce Aβ_40_ and Aβ_42_ that self-assemble through hydrophobic interactions to form oligomers, protofibrils, fibrils, and ultimately, insoluble plaques.^4–6^ It has been proposed that Aβ plaque accumulation may arise from an imbalance in Aβ production and clearance (*i.e.*, amyloid cascade hypothesis);^4,7–9^ accumulation of these peptides alone can impair neuronal mitochondrial function, leading to oxidative stress, inflammation, and the neurodegeneration commonly associated with AD (*i.e.*, oxidative stress hypothesis).^4,10–12^ In addition to self-aggregation, miscompartmentalization and dyshomeostasis of metals are found in AD-afflicted brains. In particular, elevated levels of metals, such as Cu, Zn, and Fe, are observed in Aβ plaques.^3–5,13–19^ Metal binding to Aβ is shown to facilitate peptide aggregation and in the case of redox active metal ions, reactive oxygen species (ROS) can be generated *via* Fenton-like reactions, leading to oxidative stress.^4,5,17,19–24^

On the basis of the observed metal ion dyshomeostasis, metal–Aβ interaction, and metal-involved Aβ reactivity, there has been considerable interest in the development of metal chelators capable of regulating metal ion distribution distribution and amyloid pathology. For example, the hydroxyquinoline-based antifungal drug clioquinol (CQ) decreased Aβ deposits and showed improved cognition in Phase II clinical trials for AD, in part due to its ability to inhibit binding of Zn(ii) and Cu(ii) to Aβ *via* chelation.^25–27^ Moreover, the second generation 8-hydroxyquinoline ionophore PBT2 also improved learning and memory by redistributing Cu(ii) and Zn(ii) and lowered cerebrospinal fluid levels of Aβ in Phase II clinical trials.^28,29^

Although CQ and PBT2 have presented noticeable effects on metal redistribution and Aβ clearance, the relationship between metal-associated Aβ (metal–Aβ) species and AD pathogenesis is still unclear, thus new efforts on developing chemical tools for specifically studying metal–Aβ species have been made.^21,24,30–33^ For example, the rational design of chelators containing dimethylaniline and polydentate motifs using nitrogen and oxygen donor atoms for metal ions has led to blood–brain barrier (BBB) permeable compounds that modulate metal-induced Aβ aggregation, reduce Cu–Aβ ROS formation, demonstrate antioxidant activity, and/or decrease metal–Aβ toxicity *in vitro*.^30,32–34^

Our latest approach to bifunctional chelators for Aβ modification derives from drugs such Fe-Bleomycin or hydroxyl radical footprinting reagents that act *via* Fenton chemistry and perform H-atom abstraction from the ribose ring of DNA leading to strand scission.^35,36^ Similarly, enediyne natural products such as calicheamicin that generate a potent 1,4-phenyl diradical also affect strand scission by H-atom abstraction. Radical reactions of these types however, are not limited to DNA substrates. Rather, radical-mediated footprinting is an established methodology for evaluating protein structure *via* solvent accessible reactivity,^37^ as well as for mapping protein–protein and protein–DNA interactions.^38–47^ Generation of ROS by reaction with redox active Fe, Cu, and Mn-complexes^38,48–58^ in the presence of reductant leads to controlled backbone or side chain attack which can be used to evaluate regions of macromolecular interface. While ribose ring radical strand breaks in DNA are generally due to H-atom abstraction from relatively weak tertiary C–H bonds^38^ that are statistically plentiful and readily accessible,^59^ H-atom abstraction from proteins is more complex. Direct H-abstraction from the α-carbon and side chain-assisted H-abstraction both lead to backbone cleavage,^59–61^ but poor solvent permeability and the statistical probability of extensive side chain oxidation make this process less prevalent.^37,39,46,47,59,62^ Somewhere between these limits lies calicheamicin which performs α-H abstraction from the protector protein CalC at Gly-113, cleaving the protein in a radical self-resistance mechanism.^63^

**Fig. 1:**
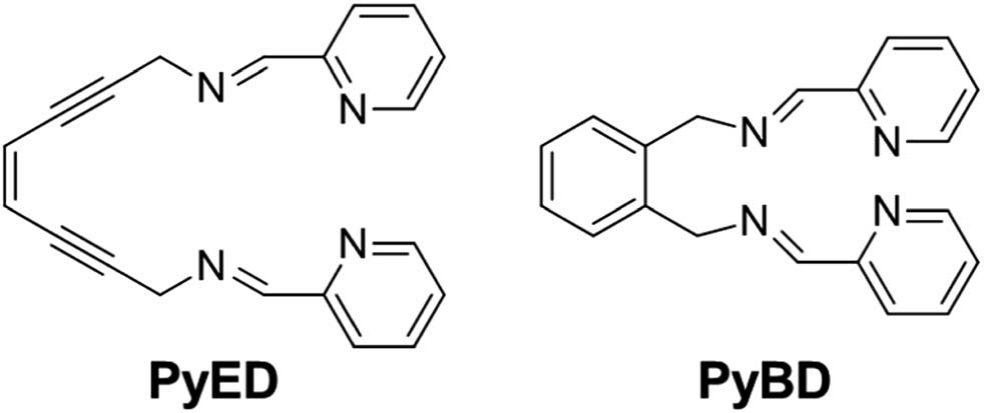
Structures of radical-generating enediyne and cyclized control ligands employed for modulation of Aβ species.

With this backdrop, we envisioned a bifunctional agent that could attack Aβ aggregates by initially chelating Aβ-bound metal ions to disrupt the peptide structure and subsequently using this chelation event to induce diradical formation that would further modify the remaining Aβ aggregates. We have shown that the compound (*Z*)-*N*,*N*′-bis[1-pyridin-2-yl-meth(*E*)-ylidene]oct-4-ene-2,6-diyne-1,8-diamine (**PyED**) (Fig. 1) binds a wide array of metal ions such as Mg,^64^ Cu, Fe, and Zn and these complexes may be thermally activated to yield a potent 1,4-diradical intermediate. Our experience with enediyne activation *via* metal coordination^64–66^ and photochemical^67–69^ diradical formation has taught us that these molecular frameworks are capable of both H-atom abstraction^64,66,67^ and addition/polymerization reactions^68,69^ depending upon the substrate and radical–radical coupling proximity. Additionally, **PyED** has demonstrated enhanced activity under clinically relevant hyperthermic conditions (42.5 °C).^70^ Although hyperthermic treatments have not commonly been applied in the field of AD, hyperthermia has been established as a method to enhance therapeutic efficiency when used in combination with other cancer treatments both *in vitro* ^71–75^ and *in vivo* ^75–78^ (≤45.5 °C). Thus, herein we report the application of such reactions to metal-bound (Cu(ii), Zn(ii)) Aβ aggregates by administration of **PyED** at physiological (37 °C) and hyperthermic (43 °C) temperatures relative to the non-radical generating control pyridine-2-ylmethyl-(2-{[(pyridine-2-ylmethylene)-amino]-methyl}-benzyl)-amine, **PyBD** (Fig. 1).

## Results and discussion

### Rationale and characterization of **PyED** and **PyBD** used for modulating metal–Aβ species

**Fig. 2:**
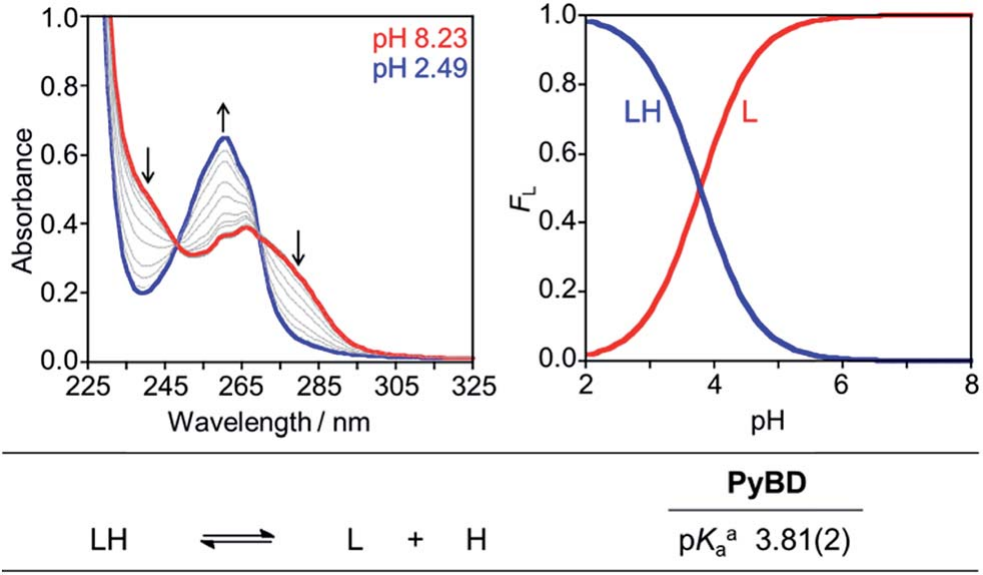
Solution speciation of **PyBD** (50 μM). Left: UV-vis spectra in the range of pH 2–8. Right: solution speciation diagram (*F*_L_ = fraction of compound in given protonation state). Bottom: acidity constants of L (L = **PyBD**) with charges omitted for clarity. Speciation was performed at room temperature with *I* = 0.1 M NaCl. ^a^Error in the last digit is indicated in parentheses.

The reactive compounds **PyED** (chelation + radical generation) and **PyBD** (chelation alone) for the modulation of Aβ species were synthesized and characterized by their ^1^H, ^13^C NMR, and mass signatures according to literature precedent.^64^ Cyclization of **PyED** by Cu(ii) or Zn(ii) chelation was investigated in MeOH and occurs within 4 h at 37 °C upon radical trapping with 1,4-cyclohexadiene and extraction with NaBH_4_ (12 equiv.)/EDTA (pH 10.6).^64^ The ^13^C NMR feature at *δ* 128 ppm and ESI-MS (*m*/*z*: 319.2) are diagnostic of cyclized product formation indicating **PyED** undergoes rapid radical formation in the presence of Cu(ii) or Zn(ii). Variable-pH UV-visible (UV-vis) titrations were conducted to evaluate the protonation state of the ligand in solution, particularly at physiologically relevant pH (pH = 7.4).^34,79,80^ In light of the fact that **PyED** slowly generates reactive radicals at ambient temperature over the timescale of the measurement (4–5 h), speciation was determined using the nonreactive, cyclized control **PyBD**. Titration results indicate a single acid ionization constant (p*K*_a_) for **PyBD** (p*K*_a_ = 3.81(2)), suggesting that neutral and monoprotonated forms of the ligand may be present in solution depending on pH. Furthermore, the solution speciation diagram reveals that **PyBD** is expected to exist mainly in the neutral form at pH 7.4 (Fig. 2).

In an effort to establish the drug-likeness of **PyED** and its potential to penetrate the BBB, Lipinski's rule parameters (MW < 450, *c* log *P* < 5.0, HBA < 10, HBD < 5) and the log BB were evaluated (Table 1).^81–84^ The resulting values (MW = 312.37, *c* log *P* = 1.01, HBA = 4, HBD = 0) indicate **PyED** has drug-like characteristics as well as possible BBB permeability (Table 1). In order to verify the predicted ability of **PyED** to penetrate the BBB, an *in vitro* PAMPA–BBB assay was performed following literature procedure.^34,81,85^ Using the empirical classification for BBB-permeable molecules, the measured permeability value, –log *P*_e_, for **PyED** (–log *P*_e_ = 4.9 ± 0.1) suggests **PyED** may be likely to penetrate the BBB.

**Table 1:** Values for Lipinski's rules and others for **PyED**

Calculation^*a*^	**PyED**	Lipinski rule parameters and others
MW	312.37	450
*c* log *P*	1.01	5.0
HBA	4	10
HBD	0	5
PSA	50.5	90 Å^2^
log BB	–0.464	0.3 (readily crosses the BBB)
		–1.0 (poorly distributed in the brain)
–log *P*_e_ ^*b*^	4.9 ± 0.1	
CNS± prediction^*c*^	CNS+	–log *P*_e_ 5.4 (CNS+)
		–log *P*_e_ 5.7 (CNS–)

^*a*^MW, molecular weight; *c* log *P*, calculated logarithm of the octanol–water partition coefficient; HBA, hydrogen-bond acceptor atoms; HBD, hydrogen-bond donor atoms; PSA, polar surface area; log BB = –0.0148 × PSA + 0.152 × *c* log *P* 0.130.

^*b*^The values of –log *P*_e_ were measured by the parallel artificial membrane permeability assay (PAMPA).

^*c*^CNS+ compounds have the ability to permeate the BBB and target the CNS, while CNS– compounds have poor permeability through the BBB and therefore, their bioavailability into the CNS is considered minimal.

### Metal binding properties of **PyED** and **PyBD**

Divalent metal binding of **PyED** and **PyBD** at 0 °C was demonstrated by bathochromic shifts of their UV-vis features (**PyED** *λ* = 264 nm; **PyBD** *λ* = 272 nm) upon addition of ZnCl_2_ or CuCl_2_ (1 equiv.) in ethanol (Fig. S1†). At 1 equiv. of MCl_2_, the absorption spectra of **PyBD** show the formation of distinct metallated species with larger bathochromic shifts observed for Cu(ii) binding relative to Zn(ii). For the more flexible chelate **PyED**, these shifts are somewhat less pronounced and indicate parallel, but slightly weaker ligand binding under these unactivating conditions (0 °C). The apparent trend of enhanced Cu(ii) binding relative to Zn(ii) is consistent with those observed for N-donor functionalities within a range of flexible ligands.^25,86–88^

**Fig. 3:**
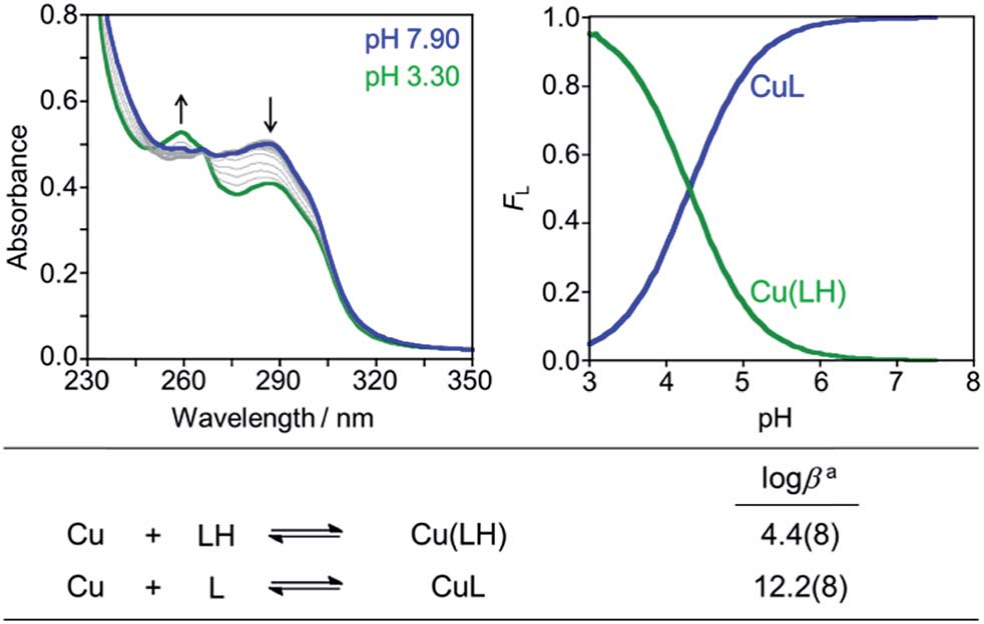
Solution speciation of the Cu(ii)–**PyBD** complex. Left: UV-vis spectra in the range of pH 3–8 ([Cu(ii)]/[**PyBD**] = 1 : 1; [Cu(ii)]_total_ = 50 μM). Right: solution speciation diagram (*F*_Cu_ = fraction of free Cu and Cu complexes). Bottom: stability constants of the Cu(ii)–**PyBD** complex with charges admitted for clarity. Titrations were performed at room temperature with *I* = 0.1 M NaCl. ^a^Error in the last digit is indicated in parentheses.

Although the neutral form of **PyBD** is the major species in solution at physiological pH (*vide supra*), variable-pH UV-vis titrations were also conducted to elucidate complexation and binding properties of **PyBD** with Cu(ii) in solution at ambient temperature and the proposed local pH for Cu(ii)–Aβ species (pH = 6.6) (Fig. 3, left). Based on the p*K*_a_ value determined for **PyBD** and these titration results, the stability constants (log *β*) for the these complexes were determined to be 12.2(8) and 4.4(8) for CuL and Cu(LH), respectively. A solution speciation diagram was modeled using these stability constants and suggests complexation of **PyBD** with Cu(ii) occurs in a 1 : 1 metal : ligand ratio. While neutral and protonated forms of Cu(ii)–**PyBD** may exist at different pH values, the data indicate that the neutral Cu(ii)–**PyBD** form is the major species at pH 6.6 (Fig. 3, right). Additionally, the concentration of free Cu(ii) in solution at pH 6.6 yields a *p*Cu value of 8.3(4) (*p*Cu = –log[Cu(ii)]_unbound_). The *p*Cu magnitude suggests an approximate *K*_d_ for Cu(ii)–**PyBD** to be *ca.* nanomolar. When considered with the reported *K*_d_ values for Cu(ii)–Aβ species (picomolar to nanomolar range),^4,5,17,19–21,24,89^ this approximate dissociation constant indicates that **PyBD** may be able to compete for Cu(ii) binding in Cu(ii)–Aβ species.

To estimate the ability of **PyED** to bind Cu(ii) in the presence of other biologically relevant metal ions such as Ca(ii), Co(ii), Fe(ii), Fe(iii), Mg(ii), Mn(ii), Ni(ii), and Zn(ii), selectivity was evaluated for the unreactive model compound **PyBD** by a competitive UV-vis absorption assay (Fig. S2†). Even in the presence of a large excess of competing metal ion, **PyBD** displays good selectivity for Cu(ii) over Ca(ii), Co(ii), Mg(ii), Mn(ii), and Ni(ii), while significant binding is shown in the presence of Fe(ii) and Fe(iii) (Fig. S2†). The observation that **PyBD** demonstrates selectivity for Cu(ii) over Zn(ii) leads to the expectation that modulation of Cu(ii)-bound Aβ species *via* metal chelation and subsequent radical generation by **PyED** may be more prominent than for the Zn(ii)-bound species (*vida infra*). Overall, the tetradentate pyridine–imine binding moiety of **PyBD** and **PyED** may be desirable for reacting with Cu(ii)–Aβ species over other biologically relevant divalent metal ions.

### Effect of PyED and PyBD on metal-free and metal-triggered Aβ aggregation *in vitro*

**Scheme 1:**
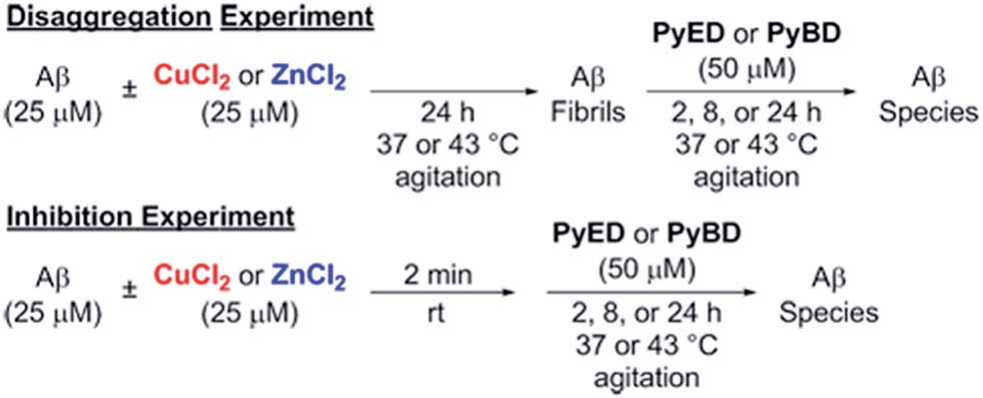
Experimental set-up for Aβ_40_ and Aβ_42_ disaggregation and inhibition assays.

**Fig. 4:**
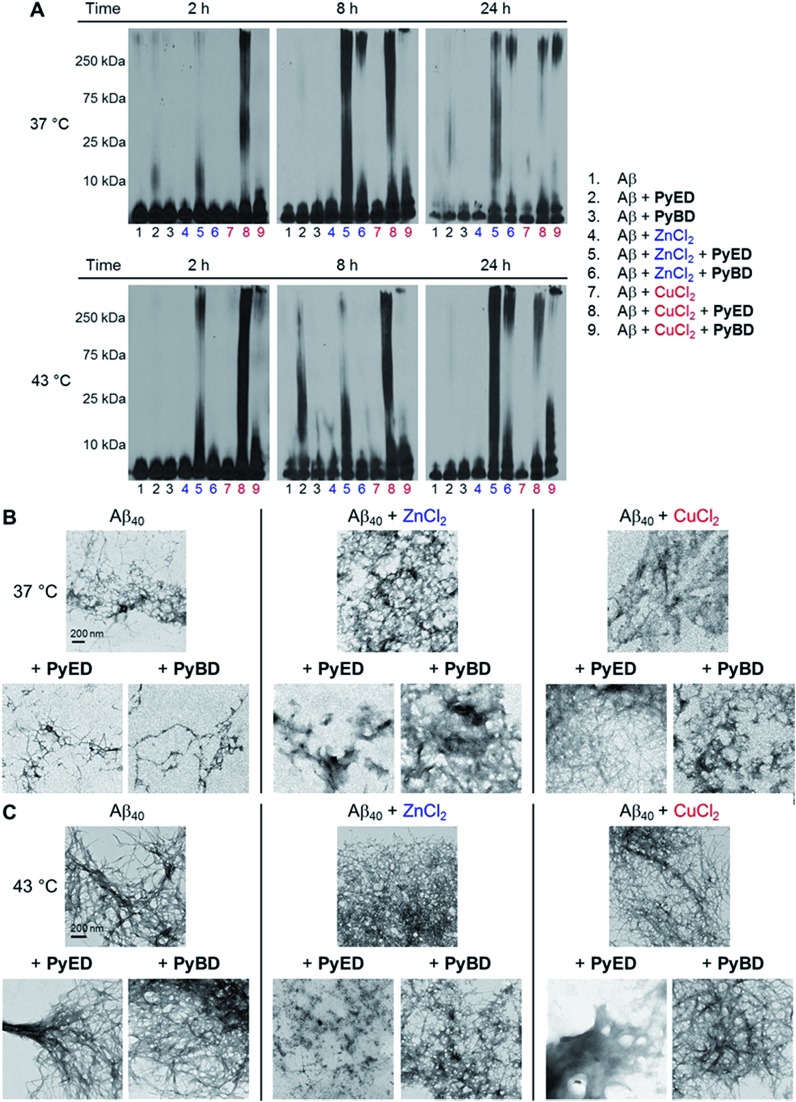
Reactivity of **PyED** and **PyBD** with preformed Aβ_40_ aggregates. (A) Analysis of resultant Aβ_40_ species by gel electrophoresis with Western blot using an anti-Aβ antibody (6E10). TEM images of the samples incubated for 24 h at (B) 37 °C or (C) 43 °C. Experimental conditions: [Aβ] = 25 μM; [CuCl_2_ or ZnCl_2_] = 25 μM; [**PyED** or **PyBD**] = 50 μM; 2, 8, 24 h incubation at 37 or 43 °C; pH 7.4 (metal-free and Zn(ii)) or pH 6.6 (Cu(ii)); constant agitation.

**Fig. 5:**
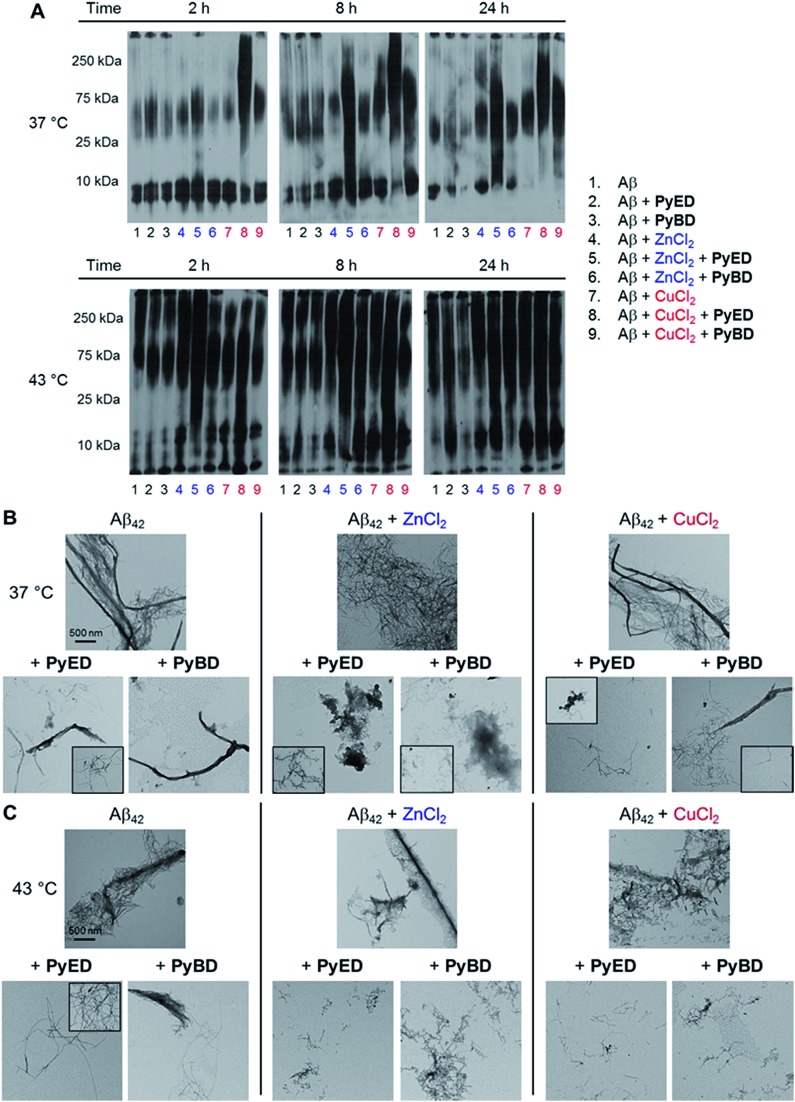
(A) Analysis of resultant Aβ_42_ species by gel electrophoresis with Western blot using an anti-Aβ antibody (6E10). TEM images of samples incubated for 24 h at (B) 37 °C or (C) 43 °C. Experimental conditions: [Aβ] = 25 μM; [CuCl_2_ or ZnCl_2_] = 25 μM; [**PyED** or **PyBD**] = 50 μM; 2, 8, 24 h incubation at 37 or 43 °C; pH 7.4 (metal-free and Zn(ii)) or pH 6.6 (Cu(ii)); constant agitation.

In order to assess the ability of bifunctional **PyED** to modulate metal-induced Aβ_40_ and Aβ_42_ aggregation pathways, *in vitro* disaggregation and inhibition experiments were conducted (Scheme 1).^32,79,80^ For comparison to **PyED** reactivity, the influence of monofunctional **PyBD** on Aβ aggregation was also examined. Disaggregation assays were designed to investigate the potential of both **PyED** and **PyBD** to structurally alter preformed metal-free and metal-associated Aβ aggregates (Fig. 4 and 5), while inhibition experiments probed the compounds' ability to control the formation of metal-free and metal-induced Aβ aggregates (Fig. S3 and S4†). The resultant Aβ species were characterized using gel electrophoresis followed by Western blot with an anti-Aβ antibody (6E10), and morphological changes were monitored by transmission electron microscopy (TEM).^32,80^

For preformed metal-free and metal-associated Aβ_40_ and Aβ_42_ aggregates, **PyED** and **PyBD** exhibit differing disaggregation capabilities (Fig. 4 and 5). In the case of Zn(ii)– and Cu(ii)–Aβ_40_ samples treated with **PyED**, Aβ species with an increasing range of molecular weights (MW) are observed at both 37 and 43 °C between 2 and 8 h, while a decrease in signal intensity occurs between 8 and 24 h for samples incubated at 37 °C (Fig. 4A, lanes 5 and 8). Interestingly, variable reactivity of **PyED** with preformed Zn(ii)–Aβ_40_ aggregates was detected at 43 °C. Addition of **PyED** presented an increasing distribution of MW throughout the time course (Fig. 4A, lane 5). In contrast, treatment of metal–Aβ_40_ samples with **PyBD** leads to the generation of lower MW species (MW ≤ 25 kDa) over the 24 h period (Fig. 4A, lanes 6 and 9). These data suggest that **PyBD** only slightly affects the transformation of preformed metal–Aβ aggregates, indicating that the introduction of radical formation upon metal binding by **PyED** may be a key factor in the generation of metal-associated Aβ species exhibiting a different array of MW. Additionally, the reduction of gel band intensities in Cu(ii)–Aβ_40_ samples incubated with **PyED** for 24 h may imply the occurrence of further aggregation over time. These results are markedly different than those observed for metal-free samples incubated with **PyED** or **PyBD** which demonstrate overall minimal disaggregation activity, with the exception of 8 h incubation under hyperthermic conditions. This modest metal-free activity is expected due to the absence of both chelation and chelation-induced radical generation pathways (Fig. 4A, lane 2). In the absence of divalent metals, initiation of thermally-induced **PyED** cyclization is slow,^64^ leading to limited radical formation under these conditions.

The trends in the gel analysis are consistent with TEM images of preformed metal–Aβ_40_ aggregates treated with **PyED**. At 37 °C, TEM images of metal–Aβ_40_ show a mixture of fibrillar and amorphous structure types, while at 43 °C amorphous Aβ morphologies are dominant. In comparison, parallel samples incubated with **PyBD** exhibit fibrillar structures similar to those under compound-free conditions at both temperatures (Fig. 4B and C). Since **PyED** and **PyBD** show minimal change in the morphology of metal-free Aβ_40_ aggregates relative to untreated samples, this suggests that variations in the fibrillar morphology may derive from chelation and chelation-induced radical mechanisms (Fig. 4B and C).

The ability of **PyED** and **PyBD** to transform preformed Aβ_42_ aggregates was also examined (Fig. 5). Relative to analogous Aβ_40_ samples, a similar trend in both **PyED** and **PyBD** reactivity with Aβ_42_ was confirmed by gel electrophoresis. Aβ_42_ species with a wide distribution of MW are observed with **PyED**-treated, metal-associated Aβ_42_ aggregates over the course of 24 h at both temperatures (Fig. 5A, lanes 5 and 8), while low reactivity is visualized in metal–Aβ_42_ samples incubated with **PyBD** (lanes 6 and 9). In the case of metal-free conditions, a slightly different ensemble of Aβ_42_ MW are produced relative to the untreated control upon addition of **PyED** or **PyBD** (lanes 1–3). In addition to these trends, the TEM images reveal that analogous to Aβ_40_, addition of **PyED** to metal–Aβ_42_ samples induces changes in the morphology of preformed aggregates. Metal-treated Aβ_42_ exposed to either **PyED** or **PyBD** show thinner fibrils of various lengths (37 and 43 °C), as well as more amorphous species (37 °C) than observed in compound-free samples (Fig. 5B and C). As demonstrated for Aβ_40_, no distinct morphology changes are observed in the metal-free Aβ_42_ samples treated with either ligand when compared to the untreated sample, indicating the importance of metal chelation in the disaggregation pathway.

In an effort to evaluate whether chelation and radical generation can influence fibril assembly, the effect of **PyED** and **PyBD** on modulation of the Aβ aggregation pathway was investigated (Fig. S3 and S4†). Upon incubation of Zn(ii) and Aβ_40_ with **PyED**, an increasing dispersion of various MW was visualized by gel analysis over prolonged exposures of up to 24 h (Fig. S3,† lane 5). In comparison, samples containing Cu(ii), Aβ_40_, and **PyED** generate different smearing patterns than those of the analogous Zn(ii)–Aβ_40_ samples (lanes 5 and 8). Similar to the disaggregation results, the gel band intensities of the Cu(ii)–Aβ_40_ samples also decrease between 8 and 24 h incubation time at both 37 and 43 °C, suggesting the possibility of further aggregation over long incubation times. The **PyED** inhibition activity compares favorably with that of **PyBD**, where only a slight modulation of the metal-induced aggregation pathway is observed (lanes 6 and 9), while exposure of metal-free Aβ_40_ to either **PyED** or **PyBD** results in little to no activity (lanes 2 and 3). Thus, analogous to the disaggregation results, these data indicate that bifunctional **PyED** exhibits greater inhibition of metal-induced Aβ aggregation compared to monofunctional **PyBD**. These findings are supported by TEM images of Aβ_40_ samples incubated at 43 °C that reveal smaller, amorphous Aβ species in the presence of divalent metal ions and **PyED** (Fig. S3C†). Importantly, no significant morphological changes are observed in the metal-free Aβ samples exposed to either **PyED** or **PyBD**. As expected, the trend in the inhibition of Aβ_42_ aggregation upon addition of **PyED** or **PyBD** is comparable to that of Aβ_40_ (Fig. S4†). Significant modification of the aggregation pathway is only visualized for metal-associated Aβ species treated with **PyED** (Fig. S4A,† lanes 5 and 8). Additionally, TEM images reveal that thinner fibrils and/or amorphous aggregates are generated in **PyED**-treated metal–Aβ_42_ samples compared to compound-free conditions (Fig. S4B and C†). Taken together, the disaggregation and inhibition results reveal the enhanced ability of **PyED** (*vs.* **PyBD**) to variably modulate metal-free and metal-induced Aβ aggregation.

From the data available on a range of molecular structures, modulation of Aβ species may derive from the differential interaction between the ligand frameworks and monomeric Aβ peptide.^4,30,32^ To evaluate the degree of this interaction, Aβ was incubated with **PyED** or control ligand **PyBD** at 0 °C for 2 h in a ratio of 6 : 1 ligand : Aβ peptide ([Aβ] = 100 μM).^30^ The resulting species were analyzed using native nanoelectrospray ionization-mass spectrometry by comparison to the established Aβ-interacting neuropeptide Leucine-enkephalin (Leu-enk).^90^ No ligand–Aβ species were detected and no significant differences were observed between the spectra obtained upon incubation with **PyED** or **PyBD**, suggesting neither ligand framework appreciably binds Aβ relative to Leu-enk.

Overall, the data suggest that the disparate capability of **PyED** to regulate Aβ aggregation is due to a combination of metal extraction from Aβ, interaction of ligand with metal-bound Aβ species, and induction of radical-mediated modification of the peptide aggregation pathway. In support of the interaction of **PyED** and **PyED** with metal-bound Aβ species at 43 °C, photo-induced loss of HCO_2_ from M(II)–Aβ disaggregation samples and subsequent loss of CO_2_ (Cu(ii)–Aβ only) on the b-ions is observed by MALDI-TOF-TOF, whereas the corresponding y-ions appear to be intact (Fig. S5 and Tables S1–S3†). Moreover, the b_7_ ion reveals that CO_2_ loss occurs N-terminal to aspartate, D7. In addition, MALDI-TOF data show that HCO_2_ loss is only operative upon incubation (4–8 h) of the components Aβ, metal ion (Cu(ii) or Zn(ii)), and ligand (**PyBD** or **PyED**) (Fig. S6–S8†). This loss of HCO_2_ from M(II)–Aβ and subsequent loss of CO_2_ (Cu(ii)–Aβ only) is also observed in the analogous 37 °C disaggregation samples (Fig. S9–S11†). Importantly, rapid addition of any of the pair of components with the same incubation time, or the mixture of three components with no incubation does not result in any detectable photo-induced loss of CO_2_. This indicates that metal and ligand must be co-localized within the metal binding consensus sequence (1–16) and is consistent with CO_2_ loss mechanisms from photo-induced redox and ESI electron capture/detachment of Cu and Zn bound peptides.^91–94^

While evidence for the interaction among Aβ, metal ions, and **PyED** or **PyBD** is suggested for the Aβ modulation pathway, and radical-induced peptide fragmentation as part of the overarching Aβ degradation process is also proposed, detection of specific, low MW fragments is more elusive. Peptidic cleavage by α-H-atom abstraction and subsequent detection of fragments by mass spectrometry is complicated by solvent accessibility and side-chain reactivity which diversify product distribution and reduce the abundances of individual species. The absence of individual peptide fragments, however, does not preclude radical damage to Aβ as all of these radical reaction pathways will lead to structural changes in metal-bound Aβ aggregates. Thus, the enhanced reactivity of **PyED** compared to **PyBD** towards metal–Aβ species is reflective of the broader role radicals may play in the modulation of overall Aβ structure.

## Conclusions

Approaches to modulate the aggregation pathway are at the forefront of current small molecule designs for AD therapy. The ensemble of existing methodologies encompasses metal chelation and interruption of peptide aggregation by ligand interaction with Aβ, as well as targeting metal–Aβ ternary complex formation as disruption mechanisms. Here we demonstrate a bifunctional approach that combines metal chelation and active radical generation to affect Aβ aggregation. Our results indicate that the ligand–metal–Aβ interaction with subsequent radical generation is a relatively rapid (2 h at 43 °C) mechanism for influencing Aβ structural integrity and thus, the aggregation pathway. This outcome may lead to new hybrid molecular constructs designed to take advantage of several Aβ interaction modes in order to achieve rapid Aβ species modulation.
