# Supplementary material for: Chelation-induced diradical formation as an approach to modulation of the amyloid-β aggregation pathway
Source: Chem Sci. 2014 Oct 30;6(2):1018–26. doi: 10.1039/c4sc01979b (PMC5811126; doi:10.1039/c4sc01979b)
Supplement: Supplementary file 1 [file SC-006-C4SC01979B-s001.pdf]

Electronic Supplementary Material (ESI) for Chemical Science.
This journal is © The Royal Society of Chemistry 2014

Supplementary Information

for

Chelation-Induced Diradical Formation as an Approach to Modulation of the Amyloid-β Aggregation Pathway

Meghan R. Porter,[a] Akiko Kochi,[bc] Jonathon A. Karty,[a] Mi Hee Lim*[cd] and Jeffrey M. Zaleski*[a]

[a]Department of Chemistry, Indiana University, Bloomington, Indiana 47405, United States

[b]Department of Chemistry, University of Michigan, Ann Arbor, Michigan 48109, United States

[c]Department of Chemistry, Ulsan National Institute of Science and Technology (UNIST), Ulsan 689-798, Korea

[d]Life Sciences Institute, University of Michigan, Ann Arbor, Michigan 48109, United States

*Corresponding authors: zaleski@indiana.edu (JMZ), mhlim@unist.ac.kr (MHL)

Figure S1. Absorption spectra of **PyED**/**PyBD** metal binding

Figure S2. Selectivity of **PyBD** for Cu(II) binding

Figure S3. Effect of **PyED** and **PyBD** on Aβ_{40} aggregate formation at 37 and 43 °C

Figure S4. Effect of **PyED** and **PyBD** on Aβ_{42} aggregate formation at 37 and 43 °C

Figure S5. MALDI-TOF-TOF mass spectra of Cu(II)–Aβ_{40} samples

Table S1. Fragment ions from post-source decay of m/z 4329 from Cu(II)−Aβ_{40}

Table S2. Fragment ions from post-source decay of m/z 4285 from Cu(II)−Aβ_{40} + **PyBD**

Table S3. Fragment ions from post-source decay of m/z 4329 from Cu(II)−Aβ_{40} + **PyBD**

Figure S6. MALDI-TOF mass spectra of Aβ_{40} samples at 43 °C

Figure S7. MALDI-TOF mass spectra of Zn(II)−Aβ_{40} samples at 43 °C

Figure S8. MALDI-TOF mass spectra of Cu(II)−Aβ_{40} samples at 43 °C

Figure S9. MALDI-TOF mass spectra of Aβ_{40} samples at 37 °C

Figure S10. MALDI-TOF mass spectra of Zn(II)–Aβ_{40} samples at 37 °C

Figure S11. MALDI-TOF mass spectra of Cu(II)–Aβ_{40} samples at 37 °C

Materials and methods

General considerations

All reagents were purchased from commercial suppliers and used as received unless otherwise stated. The ligands (*Z*)-*N*,*N'*-bis[1-pyridin-2-yl-meth-(*E*)-ylidene]oct-4-ene-2,6-diyne-1,8-diamine (**PyED**) and pyridine-2-ylmethyl-(2-{[pyridine-2-ylmethylene)-amino]-methyl}-benzyl)-amine (**PyBD**) were synthesized according to previously published procedure.[1] Aβ_{40} (DAEFRHDSGYEVHHQKLVFFAEDVGSNKGAIIGLMVGGVV) and Aβ_{42} (DAEFRHDSGYEVHHQKLVFFAEDVGSNKGAIIGLMVGGVVIA) were purchased from AnaSpec (Fremont, CA). Absorbance measurements for the Parallel Artificial Membrane Permeability Assay adapted for blood-brain barrier (PAMPA-BBB) were recorded on a SpectraMax M5 microplate reader (Molecular Devices, Sunnydale, CA). Mass spectrometry for interaction studies was conducted on a Waters Synapt G2/S mass spectrometer. MALDI-TOF spectra were recorded with a Bruker Autoflex III instrument (Bruker Daltonics, Billerica, MA) and tandem mass spectra were acquired using a 4800 TOF-TOF MALDI tandem masss spectrometer (AB Sciex, Framingham, MA). Transmission electron microscopy (TEM) images were recorded on JEOL 1010 transmission electron microscope (Electron Microscopy Center, Indiana University, Bloomington, IN) or a Philips CM-100 transmission electron microscope (Microscopy and Image Analysis Laboratory, University of Michigan, Ann Arbor, MI). Optical spectra were recorded on an Agilent 8453 UV-visible (UV-Vis) spectrophotometer.

Solution speciation determination for PyBD and the Cu(II)–PyBD complex

UV-vis variable-pH titration was used to determine the pK_a values for **PyBD**.[2] A solution (100 mM NaCl, 10 mM NaOH, pH 12) of **PyBD** (50 μM) was titrated with small aliquots of HCl and at least 30 spectra were recorded in the pH range of 2–9. Additionally, Cu(II) binding to ligand at various pH values was investigated. A solution (100 mM NaCl, 10 mM NaOH, pH 10)

containing **PyBD** (50 μM) and $CuCl_2$ (50 μM) was titrated with small aliquots of HCl and at least 30 spectra were recorded in the pH range of 3–8. The HypSpec program (Protonic Software, UK)[3] was used to calculate the acidity and stability constants and the HySS2009 program (Protonoc Software)[4] was used to model the speciation diagrams of ligand and its corresponding metal complex.

Parallel Artificial Membrane Permeability Assay adapted for the blood-brain barrier (PAMPA-BBB)

PAMPA-BBB experiments were carried out using the PAMPA Explorer kit (*p*ION Inc., Billerica, MA) using previously reported protocols with modifications.[2, 5-8] Each stock solution was diluted with Prisma HT buffer (pH 7.4, *p*ION) to a final concentration of 25 μM (1% v/v final DMSO concentration). The resulting stock solution was added to wells of the donor plate (200 μL, number of replicates = 12). The polyvinylidene fluoride (0.45 μM) filter membrane on the acceptor plate was coated with the BBB-1 lipid formulation (5 μL, *p*ION) and placed on top of the donor plate, forming a sandwich. Brain sink buffer (200 μL, *p*ION) was added to each well of the acceptor plate. The sandwich was incubated at room temperature for 4 h without stirring. The UV-vis spectra of the solutions for reference, acceptor, and donor plates were measured using a microplate reader. The PAMPA Explorer software v.3.5 (*p*ION) was used to calculate the $-\log P_e$ for each compound. Designation of CNS± was assigned by comparison to those that were identified in previous reports.[5-7]

Metal binding studies

The binding of **PyED** and **PyBD** with Cu(II) and Zn(II) was investigated by UV-vis absorption.[2] A solution of ligand (20 μM, 1% v/v DMSO) was treated with $CuCl_2$ or $ZnCl_2$ (1 equiv). Solutions were incubated at 4 °C for 1 h and the optical spectrum of the resulting solution was recorded.

Metal selectivity

The metal selectivity of **PyBD** was examined by measuring the optical changes upon addition of $CuCl_2$ (1 equiv) to a solution of **PyBD** (50 μM, 1% v/v DMSO) previously treated with another metal chloride salt (1 or 25 equiv; $MgCl_2$, $CaCl_2$, $MnCl_2$, $FeCl_2$, $FeCl_3$, $CoCl_2$, $NiCl_2$, or $ZnCl_2$).

The Fe(II) and Fe(III) solutions were prepared anaerobically and spectra recorded under nitrogen. The quantification of metal selectivity was calculated by comparing and normalizing the absorption values for the metal–ligand complexes at 285 and 305 nm to the absorption at these wavelengths before and after Cu(II) addition.

Amyloid-β peptide experiments

Amyloid-β experiments were performed according to previously published procedures with slight modifications.[2,8,9] $A\beta_{40}$ or $A\beta_{42}$ was dissolved in ammonium hydroxide (NH_4OH, 1% v/v, aq), aliquoted, lyophilized overnight, and stored at −80 °C. A stock solution was prepared by dissolving Aβ in 1% NH_4OH and diluted with ddH_2O. For the disaggregation experiment, Aβ (25 μM) with and without metal ions ($CuCl_2$ or $ZnCl_2$, 25 μM) was first incubated for 24 h at 37 or 43 °C with constant agitation. Afterward, a compound (50 μM, 1% v/v final DMSO concentration) was added and followed by an additional 2, 8, or 24 h incubation at 37 or 43 °C with constant agitation. For the inhibition experiment, Aβ (25 μM) was treated with or without metal ions ($CuCl_2$ or $ZnCl_2$; 25 μM) for 2 min, followed by addition of a compound (50 μM; 1% v/v final DMSO concentration). The resulting samples were incubated for 2, 8, or 24 h at 37 or 43 °C with constant agitation. Both studies were performed using a buffered solution (20 μM HEPES, pH 6.6 (for $CuCl_2$) or pH 7.4 (metal-free and $ZnCl_2$), 150 μM NaCl).

Gel electrophoresis

The Aβ peptide experiments described above were analyzed using gel electrophoresis followed by Western blotting with an anti-Aβ antibody (6E10, Covance, Princeton, NJ).[2, 8, 9] Each sample (10 μL) was separated using a 10–20% Tris-tricine gel (Invitrogen, Grand Island, NY). Following the separation, the proteins were transferred onto a nitrocellulose membrane and blocked with bovine serum albumin (BSA, 3% w/v, Sigma-Aldrich) in Tris-buffered saline (TBS) containing 0.1% Tween-20 (TBS-T) for 12 h ($A\beta_{40}$) or 2–3 h ($A\beta_{42}$) at ambient temperature. The membranes were incubated with an anti-Aβ antibody (1:2000) in 2% BSA (w/v in TBS-T) for 4 h at ambient temperature ($A\beta_{40}$) or overnight at 4 °C ($A\beta_{42}$). After washing, membranes were probed with the horseradish peroxidase-conjugated goat anti-mouse antibody (1:10,000) in 2% BSA for 1 h at ambient temperature. Thermo Scientific Supersignal West Pico

Chemiluminescent Substrate (Thermo Scientific, Rockford, IL) was used to visualize protein bands.

Transmission electron microscopy (TEM)

Samples for TEM were prepared following a previously reported method ($A\beta_{40}$ samples; $A\beta_{42}$ samples[2,8,9]). Glow discharge grids (Formar/Carbon 300 mesh; Electron Microscopy Sciences, Hatfield, PA) were treated with 24 h incubated samples from either disaggregation or inhibition experiments (5 μL) for 5 min ($A\beta_{40}$ samples) or 2 min ($A\beta_{42}$ samples) at room temperature and excess sample was removed with filter paper. ($A\beta_{40}$ samples): Each grid was incubated with uranyl acetate (2% w/v ddH_2O, 5 μL, 5 min). Excess uranyl acetate was blotted and the grids were washed three time with ddH_2O (5 μL) and air-dried at room temperature. Images were acquired by a JEOL 1010 transmission electron microscope (80 kV, 25,000x magnification). ($A\beta_{42}$ samples): Grids were washed with ddH_2O three times. Each grid was stained with uranyl acetate (1% w/v ddH_2O, 5 μL). Excess uranyl acetate was blotted off and grids were air-dried for 15 min at room temperature. Images were acquired by a Philips CM-100 transmission electron microscope (80 kV, 25,000x magnification).

Interaction of PyBD and PyED with Amyloid-β (Aβ) by mass spectrometry

Samples were prepared by mixing stock solutions of **PyED** or **PyBD** (300 μM in DMSO) with $A\beta_{40}$ (50 μM in 100 mM ammonium acetate buffer, pH 7) with or without $ZnCl_2$ or $CuCl_2$ (50 μM). Samples were incubated on ice for 2 h prior to analysis. Samples were dissolved in 100 mM ammonium acetate with no organic modifier and infused at 400 nL/min into a Waters Synapt G2/S mass spectrometer in sensitivity mode (resolution = 11,000) and averaged for three minutes. Samples containing Leucine-Enkephalin (Sigma, St. Louis, MO) and $A\beta_{40}$ prepared as described above were used as a positive interaction control.

Analysis of disaggregation samples by MALDI mass spectrometry

Nine microliters of each disaggregation sample were combined with of 5% v/v aqueous trifluoroacetic acid (1 μL) to disrupt fibrils and yield individual monomeric peptide. The resulting samples were desalted using a Ziptip C4 (Millipore, Billerica, MA) in accordance with

the manufacturer's instructions. Three microliters of 1:1 v/v acetonitrile:water was used to elute the peptides; the peptide-containing eluate (1 μL) was combined of 5 g/L α-cyano-4-hydroxycinnamic acid (CHCA) matrix in 50% aqueous acetonitrile, 0.1% trifluoroacetic acid (3 μL). The matrix-peptide solution (0.5 μL) was deposited onto a single spot of a stainless steel MALDI target and allowed to air dry. Initial MALDI-TOF mass spectra were recorded with a Bruker Autoflex III instrument operated in reflectron mode and externally calibrated with bovine insulin (m/z 5734), ACTH 7-38 (m/z 3660), ACTH 18-37 (m/z 2465), Glu^1-fibrinopeptide (m/z 1570), angiotensin I (m/z 1297) and des-Arg^1 bradykinin (m/z 904). Tandem mass spectra were recorded using a 4800 TOF-TOF MALDI tandem mass spectrometer; the collision cell was filled with 4×10^{-6} mbar of air and accelerating the precursor ions to 1 keV of kinetic energy with the precursor selector set to a resolving power of 150; all tandem mass spectra are the average of 5000 individual laser shots and the data were smoothed using a 5-point Gaussian function.

Notes and references

1. D. S. Rawat and J. M. Zaleski, *J. Am. Chem. Soc.,* 2001, **123**, 9675-9676.
2. J.-S. Choi, J. J. Braymer, R. P. R. Nanga, A. Ramamoorthy and M. H. Lim, *Proc. Natl. Acad. Sci. U. S. A.*, 2010, **107**, 21990-21995.
3. P. Gans, A. Sabatini and A. Vacca, *Ann. Chim.*, 1999, **89**, 45-49.
4. L. Alderighi, P. Gans, A. Ienco, D. Peters, A. Sabatini and A. Vacca, *Coord. Chem. Rev.*, 1999, **184**, 311-318.
5. *BBB Protocol and Test Compounds*; pION Inc.: Woburn, MA, 2009.
6. A. Avdeef, S. Bendels, L. Di, B. Faller, M. Kansy, K. Sugano and Y. Yamauchi, *J. Pharm. Sci.*, 2007, **96**, 2893-2909.
7. L. Di, E. H. Kerns, K. Fan, O. J. McConnell and G. T. Carter, *Eur. J. Med. Chem.*, 2003, **38**, 223-232.
8. S. Lee, X. Zheng, J. Krishnamoorthy, M. G. Savelieff, H. M. Park, J. R. Brender, J. H. Kim, J. S. Derrick, A. Kochi, H. J. Lee, C. Kim, A. Ramamoorthy, M. T. Bowers and M. H. Lim, *J. Am. Chem. Soc.*, 2014, **136**, 299-310.
9. S. S. Hindo, A. M. Mancino, J. J. Braymer, Y. Liu, S. Vivekanandan, A. Ramamoorthy and M. H. Lim, *J. Am. Chem. Soc.*, 2009, **131**, 16663-16665.

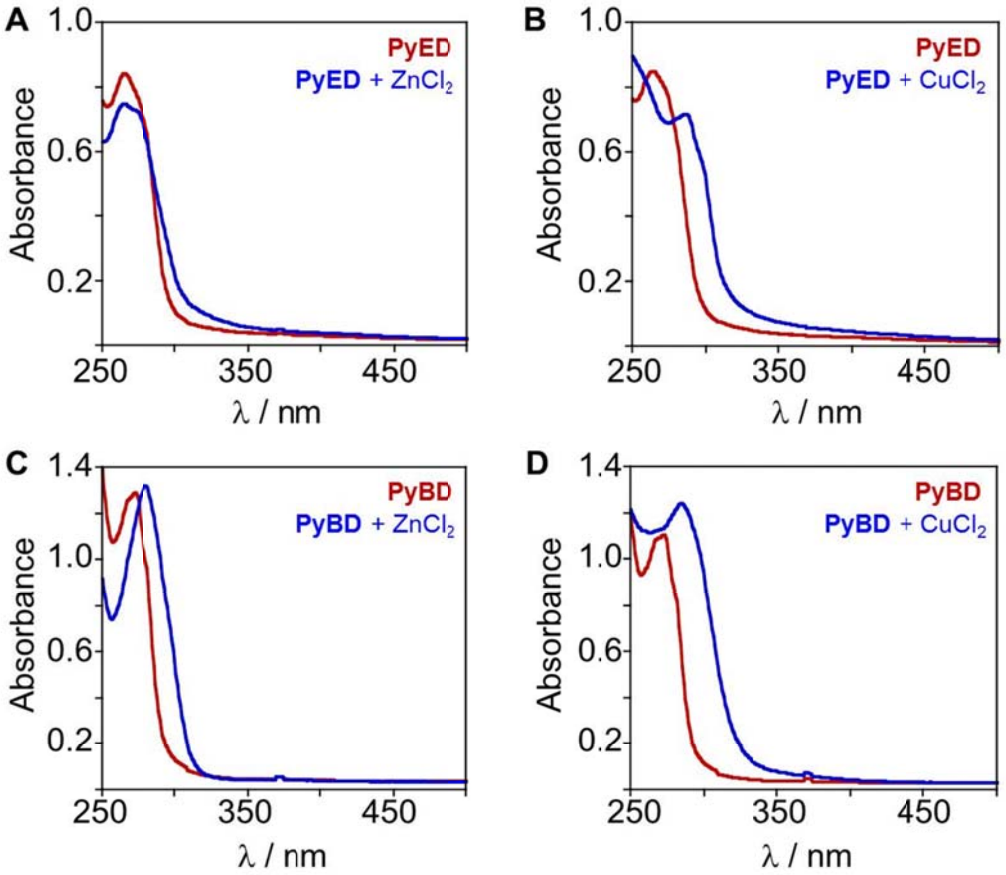

Fig. S1 UV-vis spectra showing the binding of **PyED** (A and B) and **PyBD** (C and D) with $ZnCl_2$ (A and C) and $CuCl_2$ (B and D) in ethanol. $ZnCl_2$ or $CuCl_2$ (100 μM) was added to a solution of **PyED** or **PyBD** (100 μM, red) and incubated for 10 min (blue).

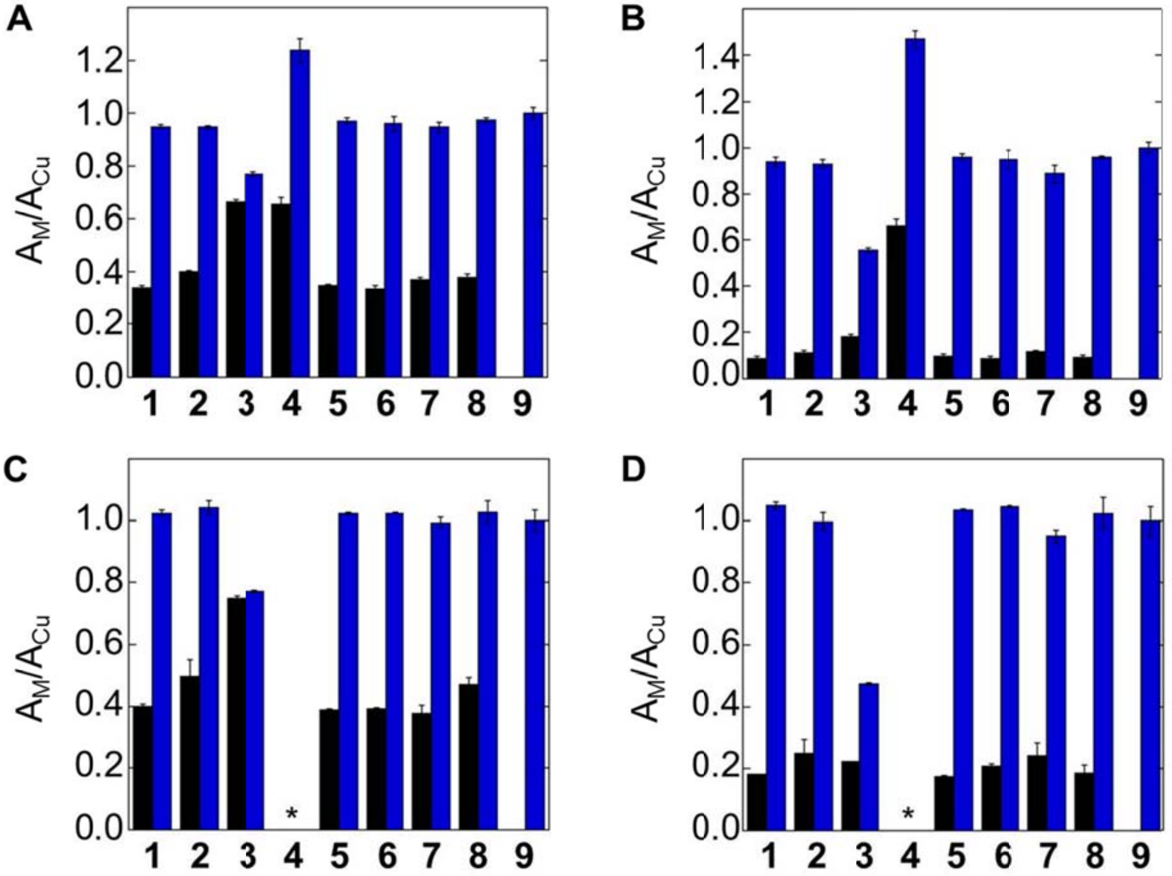

Fig. S2 Metal selectivity studies of **PyBD** (50 μM) in HEPES buffer (20 mM, pH 7.35). Blue bars indicate the addition of $CuCl_2$ (**9**) to solutions of the ligand at ambient temperature with competing metal ions (black bars: $CaCl_2$ (**1**); $CoCl_2$ (**2**); $FeCl_2$ (**3**); $FeCl_3$ (**4**); $MgCl_2$ (**5**); $MnCl_2$ (**6**); $NiCl_2$ (**7**); $ZnCl_2$ (**8**)). $CuCl_2$ addition was in a ratio of 1:1 (A and B) or 1:25 (C and D) Cu(II) to M(II) or M(III) followed by 30 min incubation at room temperature. Absorbance values at 285 (A and C) and 305 nm (B and D) were used to calculate A_M/A_{Cu} with A_{Cu} normalized to 1. Absorbance > 1 after Cu(II) addition to the ligand and competing metal solution is due to absorbance of ligand binding to the competing metal at the selected wavelength. Studies were performed at room temperature with I = 0.1 M NaCl. Precipitation observed in solution is indicated by *.

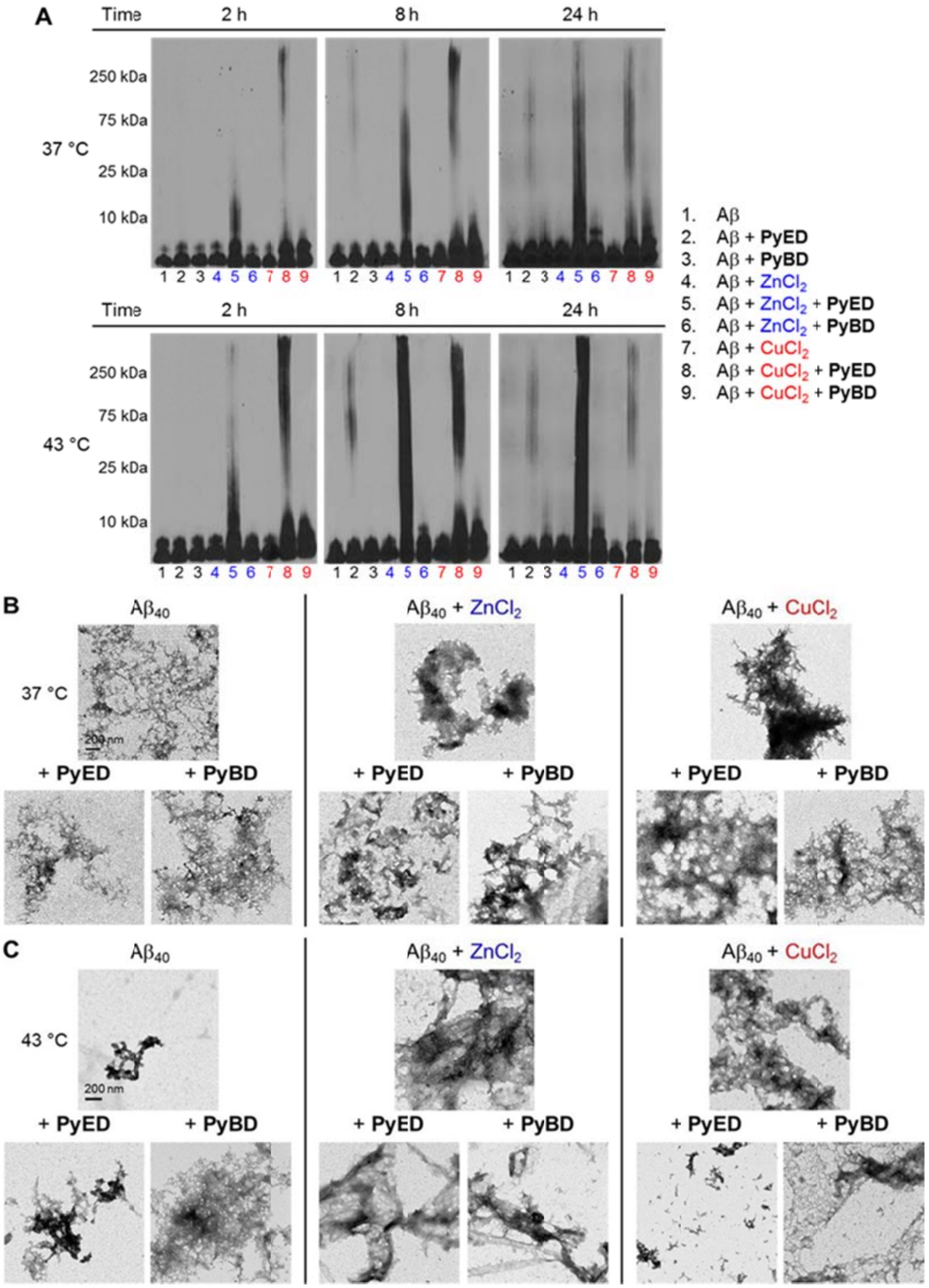

Fig. S3 Effect of **PyED** or **PyBD** on the formation of Aβ_{40} aggregates. (A) Analysis of resultant Aβ_{40} species by gel electrophoresis with Western blot using an anti-Aβ antibody (6E10). TEM images of samples incubated for 24 h at (B) 37 °C or (C) 43 °C. Experimental conditions: [Aβ] = 25 μM; [$CuCl_2$ or $ZnCl_2$] = 25 μM; [**PyED** or **PyBD**] = 50 μM; 2, 8, 24 h incubation at 37 or 43 °C; pH 7.4 (metal-free and Zn(II)) or pH 6.6 (Cu(II)); constant agitation.

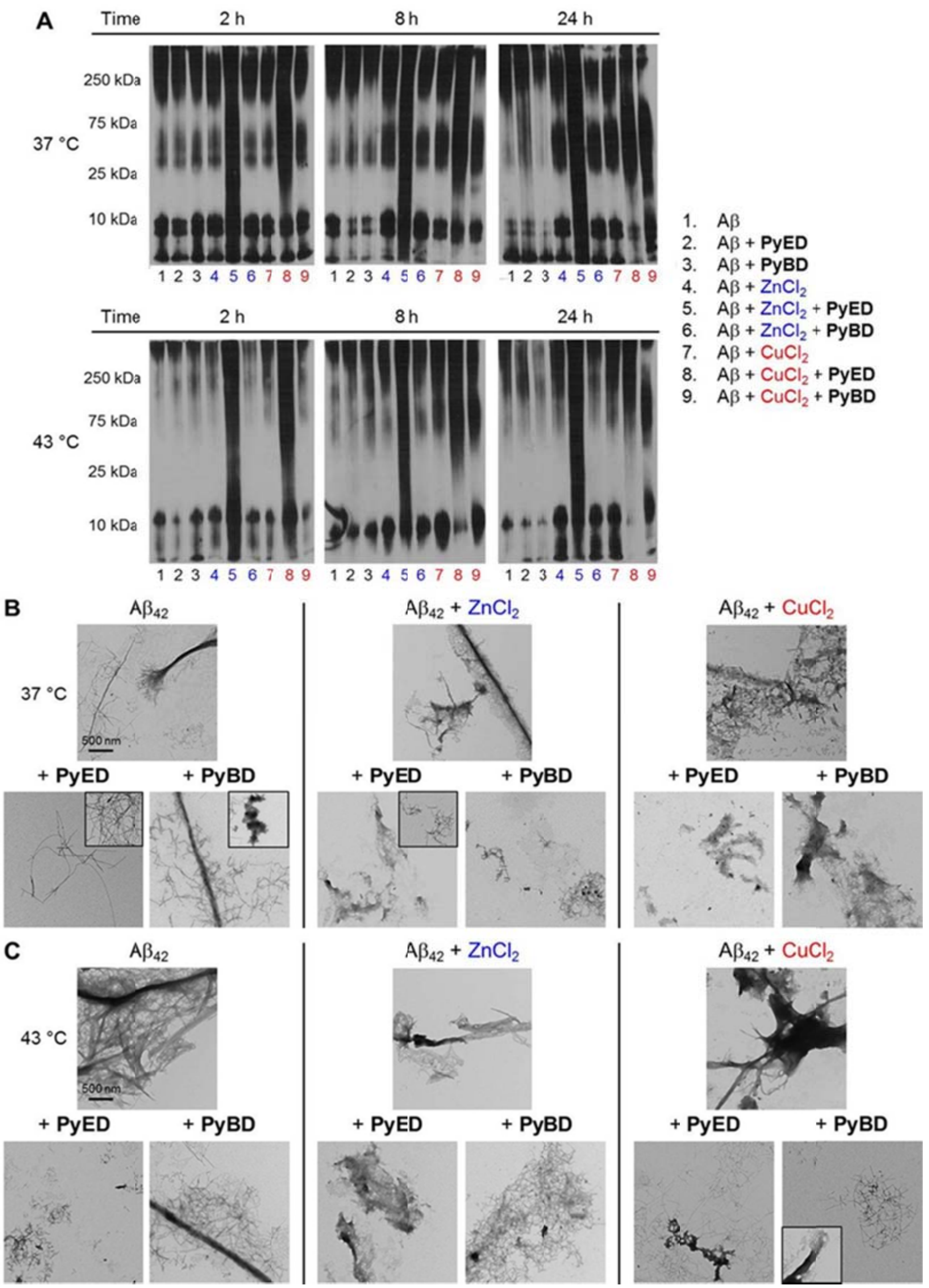

Fig. S4 (A) Analysis of resultant $A\beta_{42}$ species by gel electrophoresis with Western blot using an anti-Aβ antibody, 6E10. TEM images of samples incubated for 24 h at (B) 37 °C or (C) 43 °C. Experimental conditions: [Aβ] = 25 μM; [$CuCl_2$ or $ZnCl_2$] = 25 μM; [**PyED** or **PyBD**] = 50 μM; 2, 8, 24 h incubation at 37 or 43 °C; pH 7.4 (metal-free and Zn(II)) or pH 6.6 (Cu(II)); constant agitation.

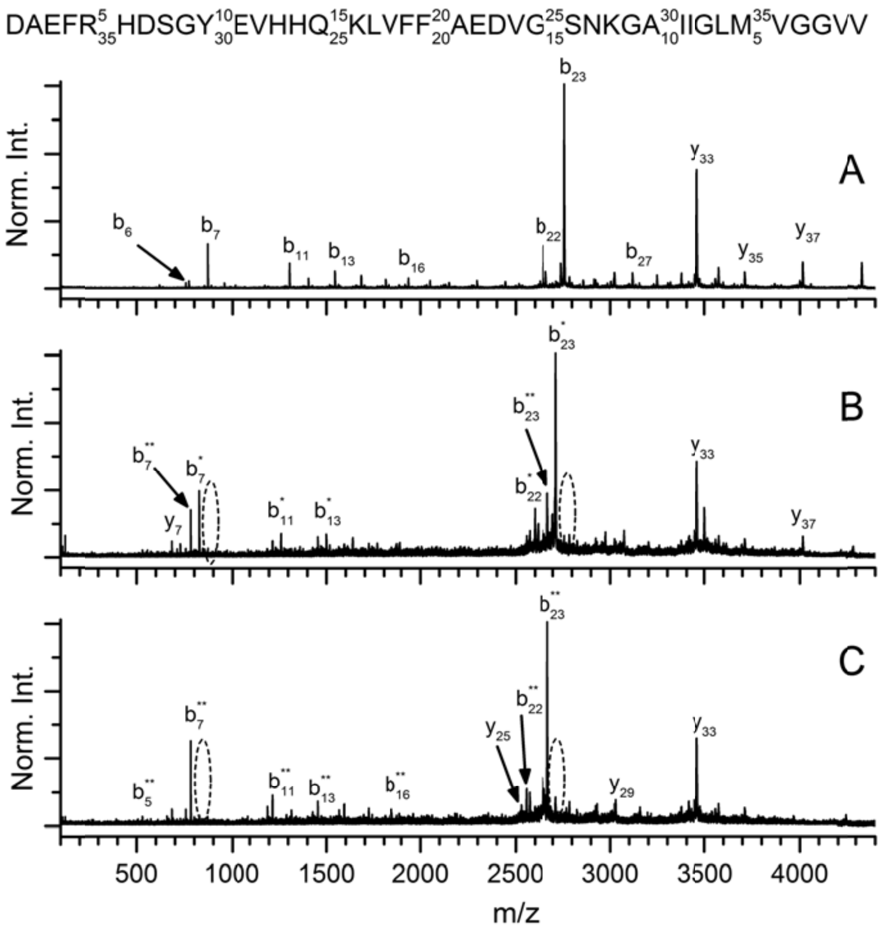

Fig. S5 ABI 4800 MALDI-TOF-TOF mass spectra for Cu(II)−Aβ_{40} disaggregation samples (4 h, 43 °C). Tandem MS of (A) m/z 4329 from Cu(II)−Aβ_{40}, (B) m/z 4285 from Cu(II)−Aβ_{40} + **PyBD**, and (C) m/z 4239 from Cu(II)−Aβ_{40} + **PyBD**. The sequence of Aβ_{40} with the locations of b-type fragmentation events labeled as superscripts and the locations of y-type fragmentations labeled as subscripts is shown above spectra. * indicates loss of 45 (HCO_2); ** indicates loss of 89 (HC_2O_4) compared to those obtained from the 4329 Da precursor.

Table S1 Fragment ions observed from post-source decay of m/z 4329 from Cu(II)–Aβ_{40} (Fig. S5A) including observed masses, relative intensities, and mass differences from predicted fragment masses.

Observed mass	Relative intensity	Likely ID	Theoretical mass	Mass difference
619.457	2.21	b_5	619.284	0.17
756.460	3.40	b_6	756.342	0.12
871.411	22.68	b_7	871.369	0.04
958.427	3.23	b_8	958.401	0.03
1015.482	2.20	b_9	1015.423	0.06
1178.451	2.11	b_{10}	1178.486	–0.04
1307.433	12.53	b_{11}	1307.529	–0.10
1406.525	5.35	b_{12}	1406.597	–0.07
1543.546	9.29	b_{13}	1543.656	–0.11
1570.822	1.70	y_{17}	1570.899	–0.08
1680.698	6.83	b_{14}	1680.715	–0.02
1808.695	4.79	b_{15}	1808.774	–0.08
1936.955	3.76	b_{16}	1936.869	0.09
2049.939	3.33	b_{17}	2049.953	–0.01
2148.876	3.16	b_{18}	2149.021	–0.15
2296.209	4.46	b_{19}	2296.089	0.12
2444.432	3.74	b_{20}	2444.688	–0.26
2644.382	20.22	b_{21}	2644.241	0.14
2759.483	94.16	b_{23}	2759.268	0.22
2858.308	4.11	b_{24}	2858.336	–0.03
2915.392	4.53	b_{25}	2915.358	0.03
3117.500	7.08	b_{27}	3117.341	0.16
3315.791	2.82	y_{31}	3315.886	–0.10
3459.912	56.97	y_{33}	3460.017	–0.11
3574.836	9.16	y_{34}	3575.106	–0.27
3599.710	3.01	b_{32}	3599.969	–0.26
3656.809	2.59	b_{33}	3657.021	–0.21
3712.253	7.78	y_{35}	3712.248	0.00
4000.350	3.79	b_{36}	4000.513	–0.16
4015.633	12.68	y_{37}	4015.615	0.02
4057.442	2.48	b_{37}	4057.565	–0.12

Table S2 Fragment ions observed from post-source decay of m/z 4285 from Cu(II)–Aβ_{40} + **PyBD** (Fig. S5B) including observed masses, relative intensities, and mass differences from predicted fragment masses.

Observed mass	Relative intensity	Likely ID	Theoretical mass	Mass difference
574.504	3.10	$b_5 - HCO_2$	574.286	0.22
674.421	2.20	y_7	674.391	0.03
711.537	3.20	$b_6 - HCO_2$	711.345	0.19
712.416	3.40	$b_6 - CO_2$	712.353	0.06
731.364	2.50	y_8	731.412	–0.05
826.449	28.80	$b_7 - HCO_2$	826.372	–0.08
844.394	4.40	y_9	844.496	0.10
913.373	3.80	$b_8 - HCO_2$	913.404	0.03
970.452	2.90	$b_9 - HCO_2$	970.425	0.03
971.504	3.60	$b_9 - CO_2$	971.433	0.07
1133.52	2.40	$b_{10} - HCO_2$	1133.49	0.04
1134.52	2.90	$b_{10} - CO_2$	1134.5	0.02
1262.57	10.50	$b_{11} - HCO_2$	1262.53	0.04
1263.41	11.60	$b_{11} - CO_2$	1263.54	–0.13
1361.52	5.60	$b_{12} - HCO_2$	1361.6	–0.08
1362.55	5.00	$b_{12} - CO_2$	1362.61	–0.06
1498.6	11.20	$b_{13} - HCO_2$	1498.66	–0.06
1499.46	10.10	$b_{13} - CO_2$	1499.67	–0.20
1570.89	3.70	y_{17}	1570.9	–0.01
1635.71	9.10	$b_{14} - HCO_2$	1635.72	–0.01
1763.75	6.60	$b_{15} - HCO_2$	1763.78	–0.03
1891.91	5.90	$b_{16} - HCO_2$	1891.87	0.04
2005.25	4.70	$b_{17} - HCO_2$	2004.95	0.29
2104.04	4.70	$b_{18} - HCO_2$	2104.02	0.02
2251.15	4.30	$b_{19} - HCO_2$	2251.09	0.06
2520.36	4.30	y_{25}	2520.39	–0.03
2598.35	16.90	$b_{22} - HCO_2$	2598.24	0.11
2648.41	9.00	y_{26}	2648.45	–0.03
2713.68	86.50	$b_{23} - HCO_2$	2713.27	0.41
2785.84	10.10	y27	2785.51	0.33
2814.15	5.90	$b_{24} - HCO_2$	2814.09	0.06
2958.27	7.50	$b_{25} - HCO_2$	2958.22	0.06
3072.33	11.90	$b_{27} - HCO_2$	3072.32	0.01
3152.79	5.10	y_{30}	3152.71	0.08
3373.22	7.60	y_{32}	3372.94	0.28
3442.16	11.40	$b_{31} - HCO_2$	3441.79	0.37
3460.38	46.50	y_{33}	3460.02	0.36
3712.29	8.70	y_{35}	3712.25	0.04
3868.24	4.70	y_{36}	3868.44	–0.20
4015.09	9.10	y_{37}	4015.61	–0.53
4215.64	2.70	y_{39}	4215.81	–0.17

Table S3 Fragment ions observed from post-source decay of m/z 4239 from Cu(II)–Aβ_{40} + **PyBD** (Fig. S5C) including observed masses, relative intensities, and mass differences from predicted fragment masses.

Observed mass	Relative intensity	Likely ID	Theoretical mass	Mass difference
530.5286	4.84	$b_5 - HC_2O_4$	530.296	–0.23
667.4667	3.62	$b_6 - HC_2O_4$	667.3549	–0.11
782.4246	37.99	$b_7 - HC_2O_4$	782.3818	–0.04
844.2457	2.66	y_9	844.4961	0.25
869.463	3.34	$b_8 - HC_2O_4$	869.4139	–0.05
926.4001	3.05	$b_9 - HC_2O_4$	926.4353	0.04
1089.459	3.95	$b_{10} - HC_2O_4$	1089.499	0.04
1213.87	3.24	y_{12}	1213.734	–0.14
1218.457	14.61	$b_{11} - HC_2O_4$	1218.541	0.08
1317.528	6.94	$b_{12} - HC_2O_4$	1317.61	0.08
1454.609	12.1	$b_{13} - HC_2O_4$	1454.669	0.06
1570.836	4.07	y_{17}	1570.899	0.06
1591.619	10.43	$b_{14} - HC_2O_4$	1591.728	0.11
1685.918	2.96	y_{18}	1685.926	0.01
1719.872	6.91	$b_{15} - HC_2O_4$	1719.786	–0.09
1814.964	2.95	y_{19}	1814.968	0.00
1847.794	7.48	$b_{16} - HC_2O_4$	1847.881	0.09
1960.873	4.36	$b_{17} - HC_2O_4$	1960.965	0.09
2059.954	4.09	$b_{18} - HC_2O_4$	2060.034	0.08
2180.016	5.52	y_{22}	2180.142	0.13
2207.393	4.34	$b_{19} - HC_2O_4$	2207.102	–0.29
2353.785	4.63	$b_{20} - HC_2O_4$	2354.17	0.38
2425.501	4.38	$b_{21} - HC_2O_4$	2425.207	–0.29
2520.673	4.64	y_{25}	2520.389	–0.28
2554.565	17.47	$b_{22} - HC_2O_4$	2554.25	–0.32
2649.796	10.42	y_{26}	2650.175	0.38
2670.506	99.66	$b_{23} - HC_2O_4$	2670.945	0.44
2768.267	5.53	$b_{24} - HC_2O_4$	2768.345	0.08
2785.295	7.74	y_{27}	2785.507	0.21
2924.781	8.35	y_{38}	2924.46	–0.32
3023.272	9.27	y_{29}	3023.593	0.32
3156.52	7.43	$b_{28} - HC_2O_4$	3156.489	–0.03
3373.051	8.95	y_{32}	3372.939	–0.11
3397.561	5.6	$b_{31} - HC_2O_4$	3397.781	0.22
3460.594	42.99	y_{33}	3460.017	–0.58
3868.439	4.63	y_{34}	3868.437	0.00

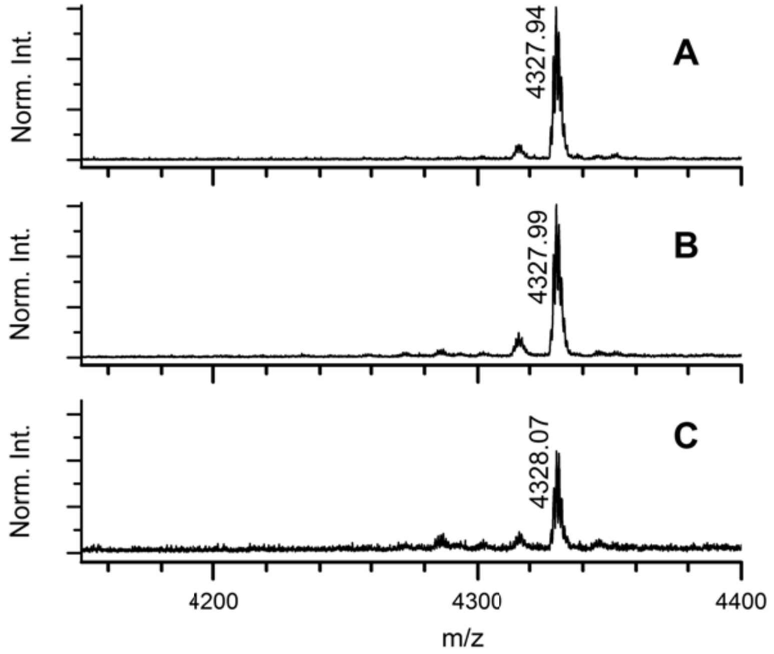

Fig. S6 MALDI-TOF mass spectra for metal free Aβ_{40} disaggregation samples (24 h, 43 °C) showing (A) Aβ only, (B) Aβ + **PyBD**, and (C) Aβ + **PyED**. The monoisotopic (all ^{12}C, ^{1}H, ^{32}S, ^{14}N, ^{16}O) peaks in each cluster are labeled.

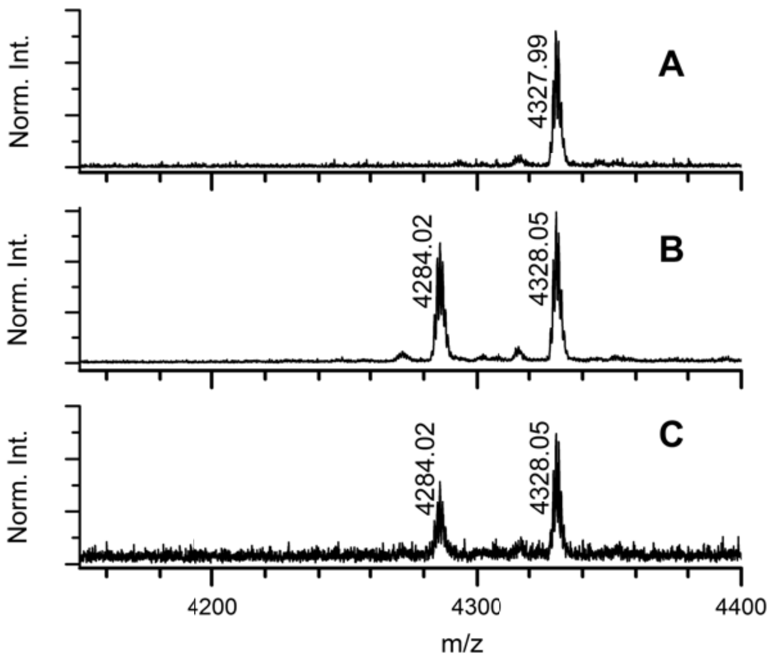

Fig. S7 MALDI-TOF mass spectra for Zn(II)−Aβ_{40} disaggregation samples (8 h, 43 °C) showing (A) Zn(II)−Aβ_{40}, (B) Zn(II)−Aβ_{40} + **PyBD**, and (C) Zn(II)−Aβ_{40} + **PyED**. The monoisotopic (all ^{12}C, ^{1}H, ^{32}S, ^{14}N, ^{16}O) peaks in each cluster are labeled.

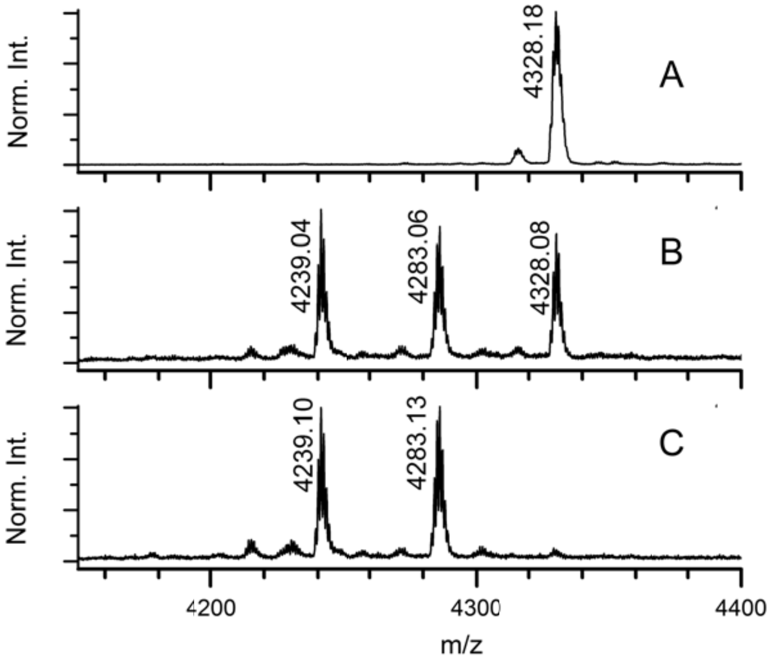

Fig. S8 MALDI-TOF mass spectra for Cu(II)−Aβ_{40} disaggregation samples (4 h, 43 °C) showing (A) Cu(II)−Aβ_{40}, (B) Cu(II)−Aβ_{40} + **PyBD**, and (C) Cu(II)−Aβ_{40} + **PyED**. The monoisotopic (all ^{12}C, ^{1}H, ^{32}S, ^{14}N, ^{16}O) peaks in each cluster are labeled.

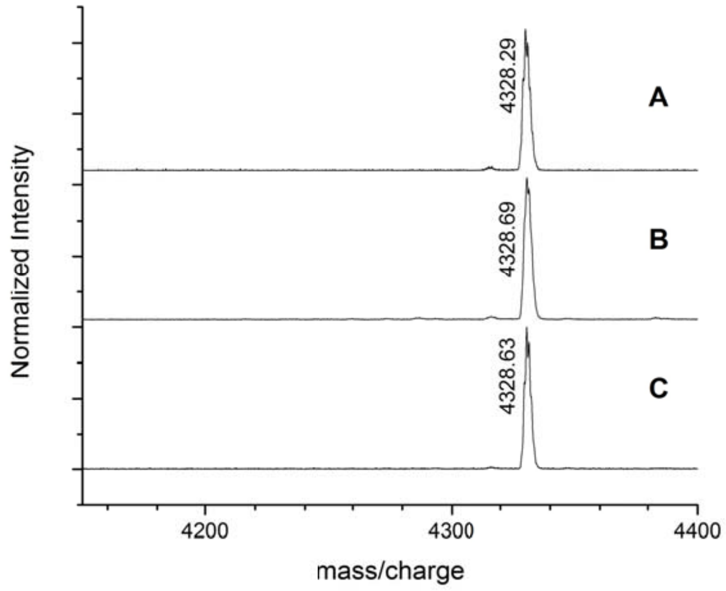

Fig. S9 MALDI-TOF mass spectra for metal free Aβ_{40} disaggregation samples (24 h, 37 °C) showing (A) Aβ only, (B) Aβ + **PyBD**, and (C) Aβ + **PyED**. The monoisotopic (all ^{12}C, ^{1}H, ^{32}S, ^{14}N, ^{16}O) peaks in each cluster are labeled.

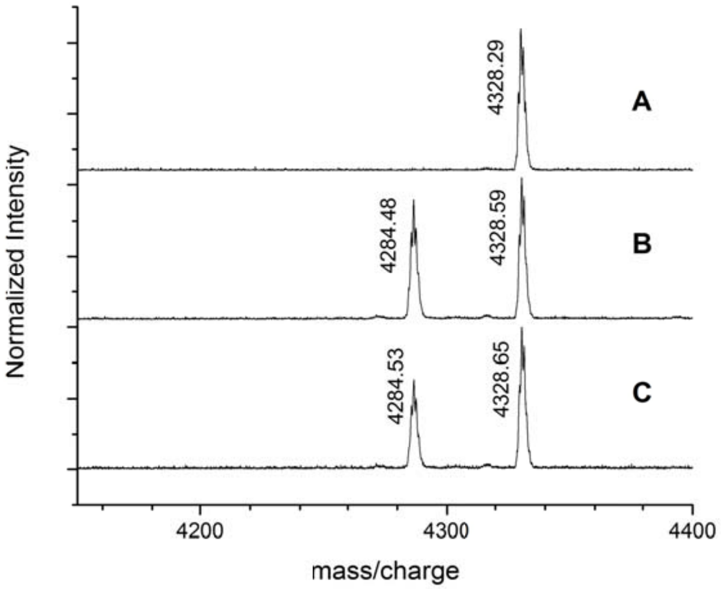

Fig. S10 MALDI-TOF mass spectra for Zn(II)−Aβ_{40} disaggregation samples (8 h, 37 °C) showing (A) Zn(II)−Aβ_{40}, (B) Zn(II)−Aβ_{40} + **PyBD**, and (C) Zn(II)−Aβ_{40} + **PyED**. The monoisotopic (all ^{12}C, ^{1}H, ^{32}S, ^{14}N, ^{16}O) peaks in each cluster are labeled.

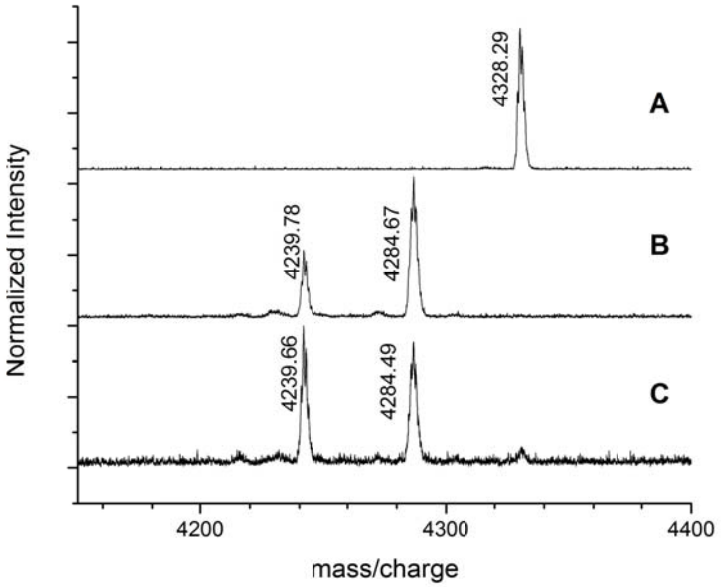

Fig. S11 MALDI-TOF mass spectra for Cu(II)−Aβ_{40} disaggregation samples (4 h, 37 °C) showing (A) Cu(II)−Aβ_{40}, (B) Cu(II)−Aβ_{40} + **PyBD**, and (C) Cu(II)−Aβ_{40} + **PyED**. The monoisotopic (all ^{12}C, ^{1}H, ^{32}S, ^{14}N, ^{16}O) peaks in each cluster are labeled.
